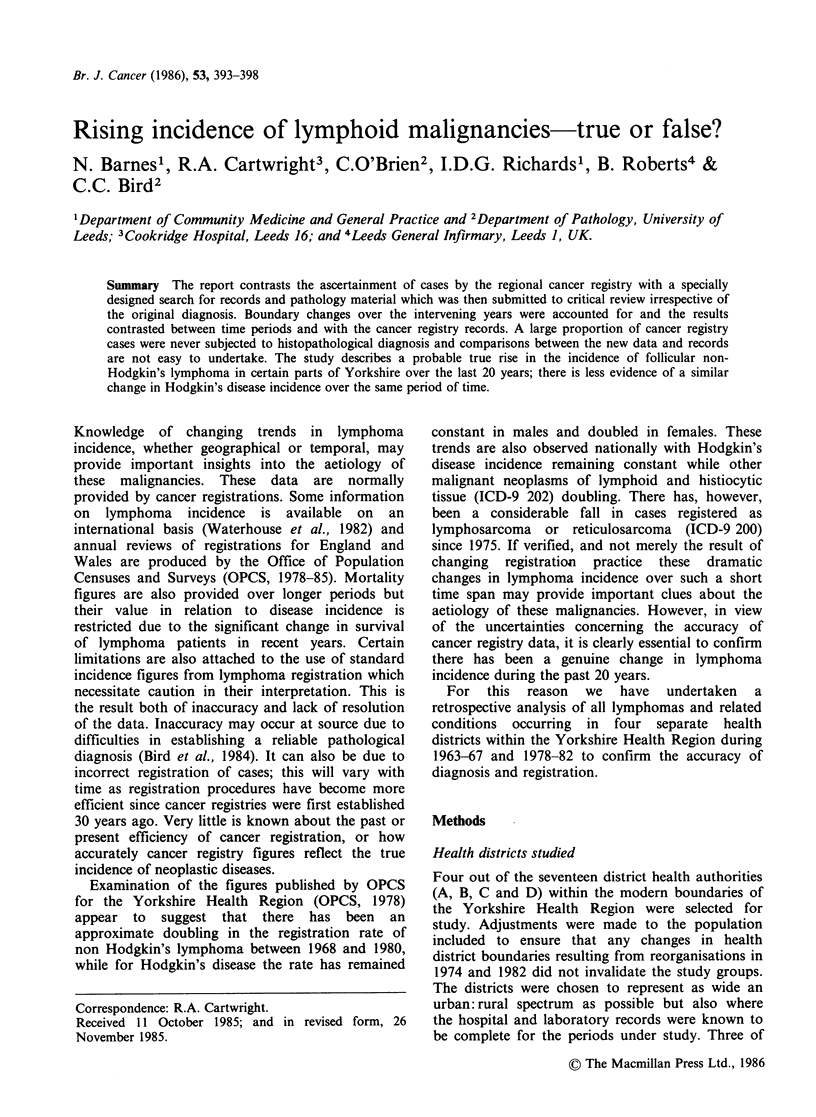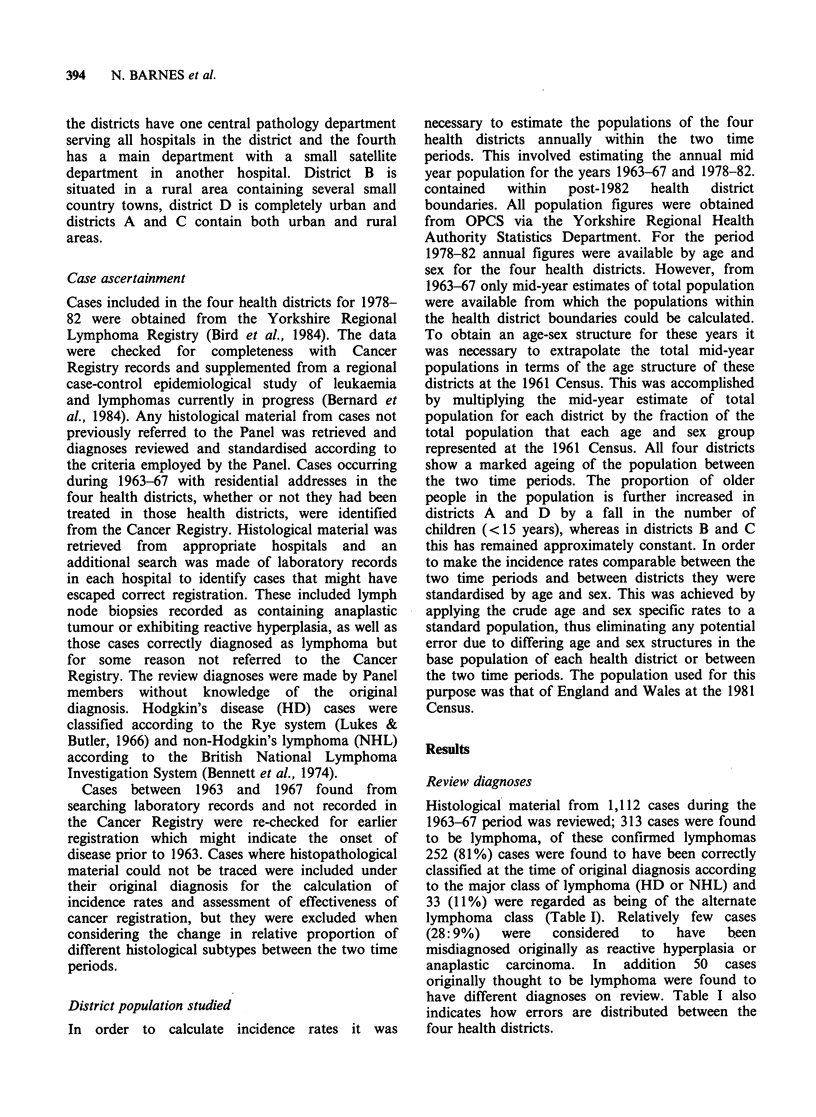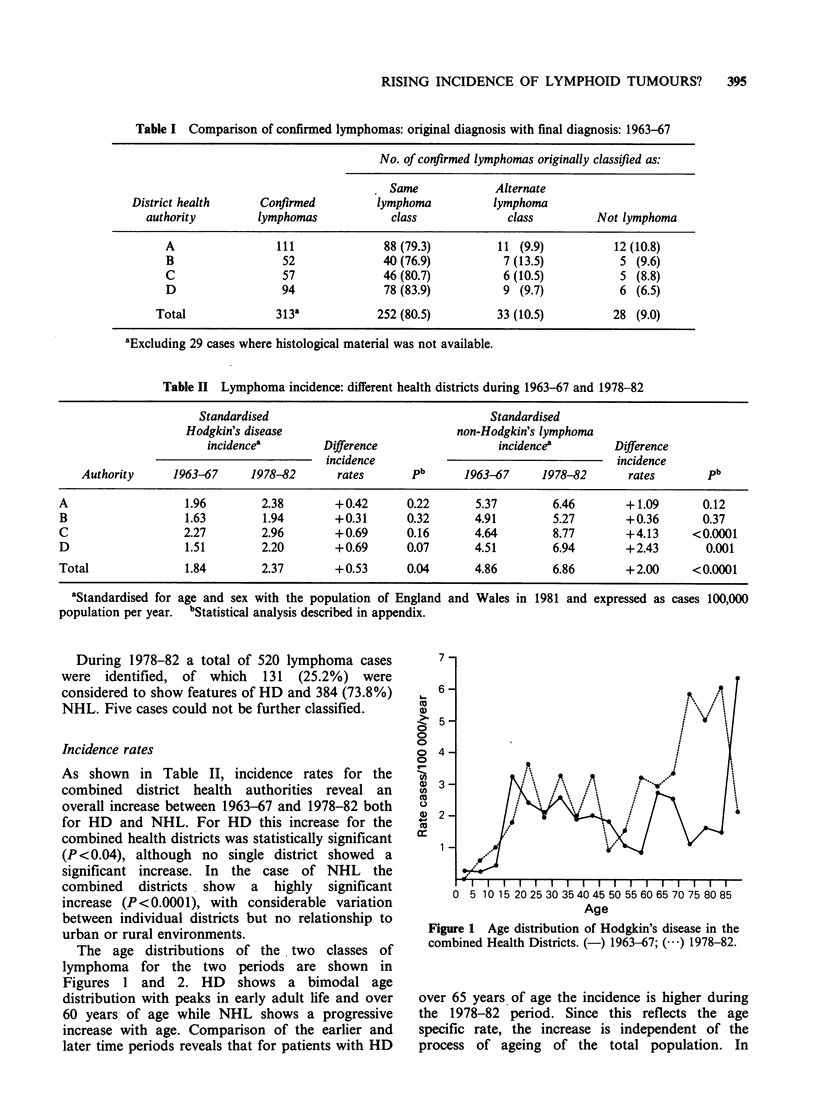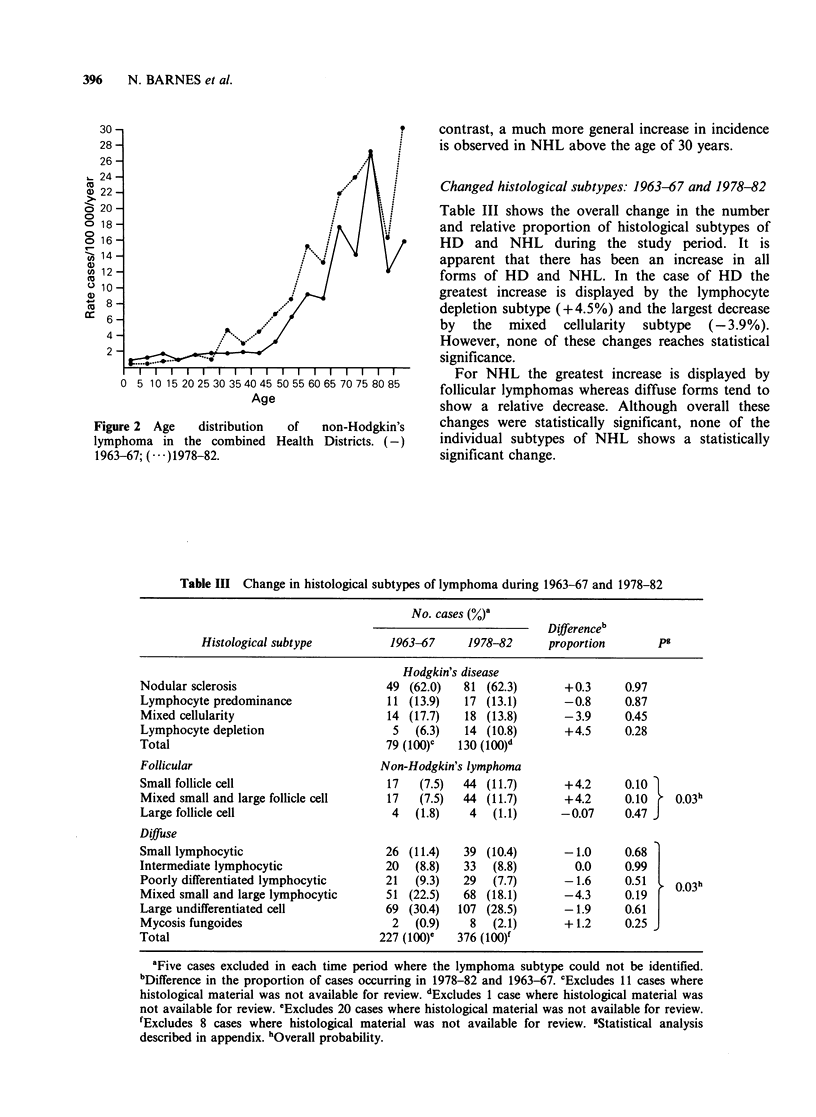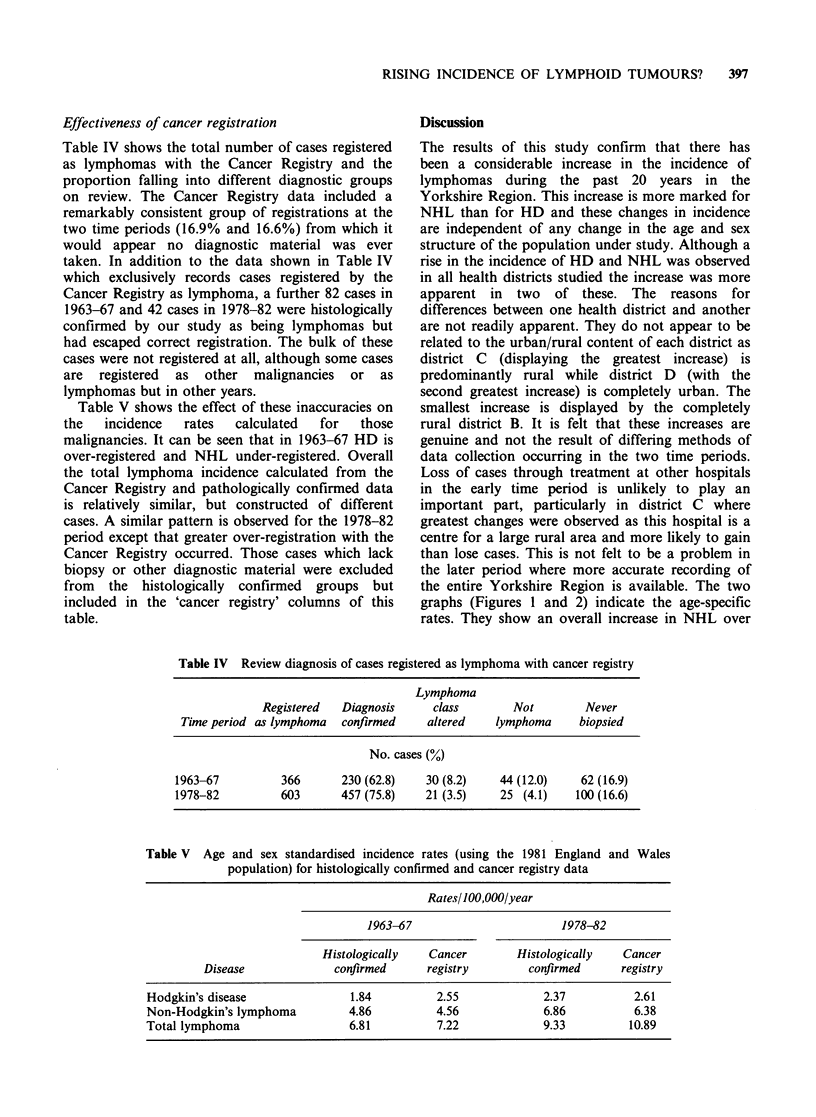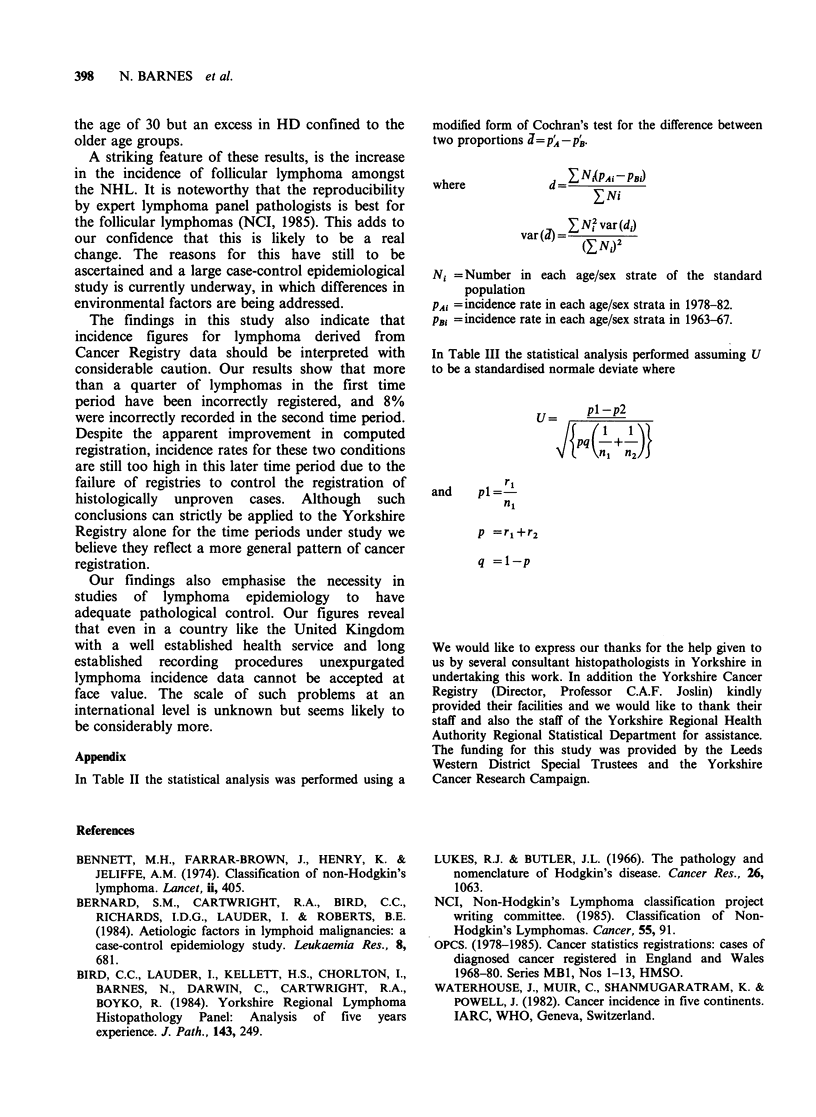# Rising incidence of lymphoid malignancies--true or false?

**DOI:** 10.1038/bjc.1986.64

**Published:** 1986-03

**Authors:** N. Barnes, R. A. Cartwright, C. O'Brien, I. D. Richards, B. Roberts, C. C. Bird

## Abstract

The report contrasts the ascertainment of cases by the regional cancer registry with a specially designed search for records and pathology material which was then submitted to critical review irrespective of the original diagnosis. Boundary changes over the intervening years were accounted for and the results contrasted between time periods and with the cancer registry records. A large proportion of cancer registry cases were never subjected to histopathological diagnosis and comparisons between the new data and records are not easy to undertake. The study describes a probable true rise in the incidence of follicular non-Hodgkin's lymphoma in certain parts of Yorkshire over the last 20 years; there is less evidence of a similar change in Hodgkin's disease incidence over the same period of time.


*Br. J. Cancer* (1986), **53**, 393–398

### Rising incidence of lymphoid malignancies—true or false?

N. Barnes[1], R.A. Cartwright[3], C.O'Brien[2], I.D.G. Richards[1], B. Roberts[4] & C.C. Bird[2]

[1]*Department of Community Medicine and General Practice and* [2]*Department of Pathology, University of Leeds;* [3]*Cookridge Hospital, Leeds 16; and* [4]*Leeds General Infirmary, Leeds 1, UK.*

**Summary** The report contrasts the ascertainment of cases by the regional cancer registry with a specially designed search for records and pathology material which was then submitted to critical review irrespective of the original diagnosis. Boundary changes over the intervening years were accounted for and the results contrasted between time periods and with the cancer registry records. A large proportion of cancer registry cases were never subjected to histopathological diagnosis and comparisons between the new data and records are not easy to undertake. The study describes a probable true rise in the incidence of follicular non-Hodgkin's lymphoma in certain parts of Yorkshire over the last 20 years; there is less evidence of a similar change in Hodgkin's disease incidence over the same period of time.

Knowledge of changing trends in lymphoma incidence, whether geographical or temporal, may provide important insights into the aetiology of these malignancies. These data are normally provided by cancer registrations. Some information on lymphoma incidence is available on an international basis (Waterhouse *et al.*, 1982) and annual reviews of registrations for England and Wales are produced by the Office of Population Censuses and Surveys (OPCS, 1978–85). Mortality figures are also provided over longer periods but their value in relation to disease incidence is restricted due to the significant change in survival of lymphoma patients in recent years. Certain limitations are also attached to the use of standard incidence figures from lymphoma registration which necessitate caution in their interpretation. This is the result both of inaccuracy and lack of resolution of the data. Inaccuracy may occur at source due to difficulties in establishing a reliable pathological diagnosis (Bird *et al.*, 1984). It can also be due to incorrect registration of cases; this will vary with time as registration procedures have become more efficient since cancer registries were first established 30 years ago. Very little is known about the past or present efficiency of cancer registration, or how accurately cancer registry figures reflect the true incidence of neoplastic diseases.

Examination of the figures published by OPCS for the Yorkshire Health Region (OPCS, 1978) appear to suggest that there has been an approximate doubling in the registration rate of non Hodgkin's lymphoma between 1968 and 1980, while for Hodgkin's disease the rate has remained constant in males and doubled in females. These trends are also observed nationally with Hodgkin's disease incidence remaining constant while other malignant neoplasms of lymphoid and histiocytic tissue (ICD-9 202) doubling. There has, however, been a considerable fall in cases registered as lymphosarcoma or reticulosarcoma (ICD-9 200) since 1975. If verified, and not merely the result of changing registration practice these dramatic changes in lymphoma incidence over such a short time span may provide important clues about the aetiology of these malignancies. However, in view of the uncertainties concerning the accuracy of cancer registry data, it is clearly essential to confirm there has been a genuine change in lymphoma incidence during the past 20 years.

For this reason we have undertaken a retrospective analysis of all lymphomas and related conditions occurring in four separate health districts within the Yorkshire Health Region during 1963–67 and 1978–82 to confirm the accuracy of diagnosis and registration.

### Methods

### *Health districts studied*

Four out of the seventeen district health authorities (A, B, C and D) within the modern boundaries of the Yorkshire Health Region were selected for study. Adjustments were made to the population included to ensure that any changes in health district boundaries resulting from reorganisations in 1974 and 1982 did not invalidate the study groups. The districts were chosen to represent as wide an urban: rural spectrum as possible but also where the hospital and laboratory records were known to be complete for the periods under study. Three of

Correspondence: R.A. Cartwright.
Received 11 October 1985; and in revised form, 26 November 1985.

© The Macmillan Press Ltd., 1986

the districts have one central pathology department serving all hospitals in the district and the fourth has a main department with a small satellite department in another hospital. District B is situated in a rural area containing several small country towns, district D is completely urban and districts A and C contain both urban and rural areas.

### Case ascertainment

Cases included in the four health districts for 1978–82 were obtained from the Yorkshire Regional Lymphoma Registry (Bird *et al.*, 1984). The data were checked for completeness with Cancer Registry records and supplemented from a regional case-control epidemiological study of leukaemia and lymphomas currently in progress (Bernard *et al.*, 1984). Any histological material from cases not previously referred to the Panel was retrieved and diagnoses reviewed and standardised according to the criteria employed by the Panel. Cases occurring during 1963–67 with residential addresses in the four health districts, whether or not they had been treated in those health districts, were identified from the Cancer Registry. Histological material was retrieved from appropriate hospitals and an additional search was made of laboratory records in each hospital to identify cases that might have escaped correct registration. These included lymph node biopsies recorded as containing anaplastic tumour or exhibiting reactive hyperplasia, as well as those cases correctly diagnosed as lymphoma but for some reason not referred to the Cancer Registry. The review diagnoses were made by Panel members without knowledge of the original diagnosis. Hodgkin's disease (HD) cases were classified according to the Rye system (Lukes & Butler, 1966) and non-Hodgkin's lymphoma (NHL) according to the British National Lymphoma Investigation System (Bennett *et al.*, 1974).

Cases between 1963 and 1967 found from searching laboratory records and not recorded in the Cancer Registry were re-checked for earlier registration which might indicate the onset of disease prior to 1963. Cases where histopathological material could not be traced were included under their original diagnosis for the calculation of incidence rates and assessment of effectiveness of cancer registration, but they were excluded when considering the change in relative proportion of different histological subtypes between the two time periods.

### District population studied

In order to calculate incidence rates it was necessary to estimate the populations of the four health districts annually within the two time periods. This involved estimating the annual mid year population for the years 1963–67 and 1978–82. contained within post-1982 health district boundaries. All population figures were obtained from OPCS via the Yorkshire Regional Health Authority Statistics Department. For the period 1978–82 annual figures were available by age and sex for the four health districts. However, from 1963–67 only mid-year estimates of total population were available from which the populations within the health district boundaries could be calculated. To obtain an age-sex structure for these years it was necessary to extrapolate the total mid-year populations in terms of the age structure of these districts at the 1961 Census. This was accomplished by multiplying the mid-year estimate of total population for each district by the fraction of the total population that each age and sex group represented at the 1961 Census. All four districts show a marked ageing of the population between the two time periods. The proportion of older people in the population is further increased in districts A and D by a fall in the number of children (<15 years), whereas in districts B and C this has remained approximately constant. In order to make the incidence rates comparable between the two time periods and between districts they were standardised by age and sex. This was achieved by applying the crude age and sex specific rates to a standard population, thus eliminating any potential error due to differing age and sex structures in the base population of each health district or between the two time periods. The population used for this purpose was that of England and Wales at the 1981 Census.

### Results

### Review diagnoses

Histological material from 1,112 cases during the 1963–67 period was reviewed; 313 cases were found to be lymphoma, of these confirmed lymphomas 252 (81%) cases were found to have been correctly classified at the time of original diagnosis according to the major class of lymphoma (HD or NHL) and 33 (11%) were regarded as being of the alternate lymphoma class (Table I). Relatively few cases (28:9%) were considered to have been misdiagnosed originally as reactive hyperplasia or anaplastic carcinoma. In addition 50 cases originally thought to be lymphoma were found to have different diagnoses on review. Table I also indicates how errors are distributed between the four health districts.

**Table I** Comparison of confirmed lymphomas: original diagnosis with final diagnosis: 1963–67

| District health authority | Confirmed lymphomas | *No. of confirmed lymphomas originally classified as:* Same lymphoma class | Alternate lymphoma class | Not lymphoma |
|---|---|---|---|---|
| A | 111 | 88 (79.3) | 11 (9.9) | 12 (10.8) |
| B | 52 | 40 (76.9) | 7 (13.5) | 5 (9.6) |
| C | 57 | 46 (80.7) | 6 (10.5) | 5 (8.8) |
| D | 94 | 78 (83.9) | 9 (9.7) | 6 (6.5) |
| Total | 313[a] | 252 (80.5) | 33 (10.5) | 28 (9.0) |

[a]Excluding 29 cases where histological material was not available.

**Table II** Lymphoma incidence: different health districts during 1963–67 and 1978–82

| Authority | *Standardised Hodgkin's disease incidence*[a] 1963–67 | 1978–82 | *Difference incidence rates* | *P*[b] | *Standardised non-Hodgkin's lymphoma incidence*[a] 1963–67 | 1978–82 | *Difference incidence rates* | *P*[b] |
|---|---|---|---|---|---|---|---|---|
| A | 1.96 | 2.38 | +0.42 | 0.22 | 5.37 | 6.46 | +1.09 | 0.12 |
| B | 1.63 | 1.94 | +0.31 | 0.32 | 4.91 | 5.27 | +0.36 | 0.37 |
| C | 2.27 | 2.96 | +0.69 | 0.16 | 4.64 | 8.77 | +4.13 | <0.0001 |
| D | 1.51 | 2.20 | +0.69 | 0.07 | 4.51 | 6.94 | +2.43 | 0.001 |
| Total | 1.84 | 2.37 | +0.53 | 0.04 | 4.86 | 6.86 | +2.00 | <0.0001 |

[a]Standardised for age and sex with the population of England and Wales in 1981 and expressed as cases 100,000 population per year. [b]Statistical analysis described in appendix.

During 1978–82 a total of 520 lymphoma cases were identified, of which 131 (25.2%) were considered to show features of HD and 384 (73.8%) NHL. Five cases could not be further classified.

### *Incidence rates*

As shown in Table II, incidence rates for the combined district health authorities reveal an overall increase between 1963–67 and 1978–82 both for HD and NHL. For HD this increase for the combined health districts was statistically significant ($P<0.04$), although no single district showed a significant increase. In the case of NHL the combined districts show a highly significant increase ($P<0.0001$), with considerable variation between individual districts but no relationship to urban or rural environments.

The age distributions of the two classes of lymphoma for the two periods are shown in Figures 1 and 2. HD shows a bimodal age distribution with peaks in early adult life and over 60 years of age while NHL shows a progressive increase with age. Comparison of the earlier and later time periods reveals that for patients with HD

**Figure 1** Age distribution of Hodgkin's disease in the combined Health Districts. (—) 1963–67; (···) 1978–82.

over 65 years of age the incidence is higher during the 1978–82 period. Since this reflects the age specific rate, the increase is independent of the process of ageing of the total population. In

**Figure 2** Age distribution of non-Hodgkin's lymphoma in the combined Health Districts. (–) 1963–67; (···)1978–82.

contrast, a much more general increase in incidence is observed in NHL above the age of 30 years.

*Changed histological subtypes: 1963–67 and 1978–82*

Table III shows the overall change in the number and relative proportion of histological subtypes of HD and NHL during the study period. It is apparent that there has been an increase in all forms of HD and NHL. In the case of HD the greatest increase is displayed by the lymphocyte depletion subtype (+4.5%) and the largest decrease by the mixed cellularity subtype (−3.9%). However, none of these changes reaches statistical significance.

For NHL the greatest increase is displayed by follicular lymphomas whereas diffuse forms tend to show a relative decrease. Although overall these changes were statistically significant, none of the individual subtypes of NHL shows a statistically significant change.

**Table III** Change in histological subtypes of lymphoma during 1963–67 and 1978–82

| *Histological subtype* | *No. cases (%)*[a] *1963–67* | *1978–82* | *Difference*[b] *proportion* | *P*[g] | |
|---|---|---|---|---|---|
| | *Hodgkin's disease* | | | | |
| Nodular sclerosis | 49 (62.0) | 81 (62.3) | +0.3 | 0.97 | |
| Lymphocyte predominance | 11 (13.9) | 17 (13.1) | −0.8 | 0.87 | |
| Mixed cellularity | 14 (17.7) | 18 (13.8) | −3.9 | 0.45 | |
| Lymphocyte depletion | 5 (6.3) | 14 (10.8) | +4.5 | 0.28 | |
| Total | 79 (100)[c] | 130 (100)[d] | | | |
| *Follicular* | *Non-Hodgkin's lymphoma* | | | | |
| Small follicle cell | 17 (7.5) | 44 (11.7) | +4.2 | 0.10 | 0.03[h] |
| Mixed small and large follicle cell | 17 (7.5) | 44 (11.7) | +4.2 | 0.10 | |
| Large follicle cell | 4 (1.8) | 4 (1.1) | −0.07 | 0.47 | |
| *Diffuse* | | | | | |
| Small lymphocytic | 26 (11.4) | 39 (10.4) | −1.0 | 0.68 | 0.03[h] |
| Intermediate lymphocytic | 20 (8.8) | 33 (8.8) | 0.0 | 0.99 | |
| Poorly differentiated lymphocytic | 21 (9.3) | 29 (7.7) | −1.6 | 0.51 | |
| Mixed small and large lymphocytic | 51 (22.5) | 68 (18.1) | −4.3 | 0.19 | |
| Large undifferentiated cell | 69 (30.4) | 107 (28.5) | −1.9 | 0.61 | |
| Mycosis fungoides | 2 (0.9) | 8 (2.1) | +1.2 | 0.25 | |
| Total | 227 (100)[e] | 376 (100)[f] | | | |

[a]Five cases excluded in each time period where the lymphoma subtype could not be identified. [b]Difference in the proportion of cases occurring in 1978–82 and 1963–67. [c]Excludes 11 cases where histological material was not available for review. [d]Excludes 1 case where histological material was not available for review. [e]Excludes 20 cases where histological material was not available for review. [f]Excludes 8 cases where histological material was not available for review. [g]Statistical analysis described in appendix. [h]Overall probability.

*Effectiveness of cancer registration*

Table IV shows the total number of cases registered as lymphomas with the Cancer Registry and the proportion falling into different diagnostic groups on review. The Cancer Registry data included a remarkably consistent group of registrations at the two time periods (16.9% and 16.6%) from which it would appear no diagnostic material was ever taken. In addition to the data shown in Table IV which exclusively records cases registered by the Cancer Registry as lymphoma, a further 82 cases in 1963–67 and 42 cases in 1978–82 were histologically confirmed by our study as being lymphomas but had escaped correct registration. The bulk of these cases were not registered at all, although some cases are registered as other malignancies or as lymphomas but in other years.

Table V shows the effect of these inaccuracies on the incidence rates calculated for those malignancies. It can be seen that in 1963–67 HD is over-registered and NHL under-registered. Overall the total lymphoma incidence calculated from the Cancer Registry and pathologically confirmed data is relatively similar, but constructed of different cases. A similar pattern is observed for the 1978–82 period except that greater over-registration with the Cancer Registry occurred. Those cases which lack biopsy or other diagnostic material were excluded from the histologically confirmed groups but included in the 'cancer registry' columns of this table.

### Discussion

The results of this study confirm that there has been a considerable increase in the incidence of lymphomas during the past 20 years in the Yorkshire Region. This increase is more marked for NHL than for HD and these changes in incidence are independent of any change in the age and sex structure of the population under study. Although a rise in the incidence of HD and NHL was observed in all health districts studied the increase was more apparent in two of these. The reasons for differences between one health district and another are not readily apparent. They do not appear to be related to the urban/rural content of each district as district C (displaying the greatest increase) is predominantly rural while district D (with the second greatest increase) is completely urban. The smallest increase is displayed by the completely rural district B. It is felt that these increases are genuine and not the result of differing methods of data collection occurring in the two time periods. Loss of cases through treatment at other hospitals in the early time period is unlikely to play an important part, particularly in district C where greatest changes were observed as this hospital is a centre for a large rural area and more likely to gain than lose cases. This is not felt to be a problem in the later period where more accurate recording of the entire Yorkshire Region is available. The two graphs (Figures 1 and 2) indicate the age-specific rates. They show an overall increase in NHL over

**Table IV** Review diagnosis of cases registered as lymphoma with cancer registry

| *Time period* | *Registered as lymphoma* | *Diagnosis confirmed* | *Lymphoma class altered* | *Not lymphoma* | *Never biopsied* |
|---|---|---|---|---|---|
| | | No. cases (%) | | | |
| 1963–67 | 366 | 230 (62.8) | 30 (8.2) | 44 (12.0) | 62 (16.9) |
| 1978–82 | 603 | 457 (75.8) | 21 (3.5) | 25 (4.1) | 100 (16.6) |

**Table V** Age and sex standardised incidence rates (using the 1981 England and Wales population) for histologically confirmed and cancer registry data

| | *Rates/100,000/year* | | | |
|---|---|---|---|---|
| | *1963–67* | | *1978–82* | |
| *Disease* | *Histologically confirmed* | *Cancer registry* | *Histologically confirmed* | *Cancer registry* |
| Hodgkin's disease | 1.84 | 2.55 | 2.37 | 2.61 |
| Non-Hodgkin's lymphoma | 4.86 | 4.56 | 6.86 | 6.38 |
| Total lymphoma | 6.81 | 7.22 | 9.33 | 10.89 |

the age of 30 but an excess in HD confined to the older age groups.

A striking feature of these results, is the increase in the incidence of follicular lymphoma amongst the NHL. It is noteworthy that the reproducibility by expert lymphoma panel pathologists is best for the follicular lymphomas (NCI, 1985). This adds to our confidence that this is likely to be a real change. The reasons for this have still to be ascertained and a large case-control epidemiological study is currently underway, in which differences in environmental factors are being addressed.

The findings in this study also indicate that incidence figures for lymphoma derived from Cancer Registry data should be interpreted with considerable caution. Our results show that more than a quarter of lymphomas in the first time period have been incorrectly registered, and 8% were incorrectly recorded in the second time period. Despite the apparent improvement in computed registration, incidence rates for these two conditions are still too high in this later time period due to the failure of registries to control the registration of histologically unproven cases. Although such conclusions can strictly be applied to the Yorkshire Registry alone for the time periods under study we believe they reflect a more general pattern of cancer registration.

Our findings also emphasise the necessity in studies of lymphoma epidemiology to have adequate pathological control. Our figures reveal that even in a country like the United Kingdom with a well established health service and long established recording procedures unexpurgated lymphoma incidence data cannot be accepted at face value. The scale of such problems at an international level is unknown but seems likely to be considerably more.

### Appendix

In Table II the statistical analysis was performed using a modified form of Cochran's test for the difference between two proportions $\bar{d}=p'_A-p'_B$.

where
$$d=\frac{\sum N_i(p_{Ai}-p_{Bi})}{\sum Ni}$$

$$\text{var}(\bar{d})=\frac{\sum N_i^2\,\text{var}(d_i)}{(\sum N_i)^2}$$

$N_i$ =Number in each age/sex strate of the standard population
$p_{Ai}$ =incidence rate in each age/sex strata in 1978–82.
$p_{Bi}$ =incidence rate in each age/sex strata in 1963–67.

In Table III the statistical analysis performed assuming $U$ to be a standardised normale deviate where

$$U=\frac{p1-p2}{\sqrt{\left\{pq\left(\frac{1}{n_1}+\frac{1}{n_2}\right)\right\}}}$$

and $p1=\frac{r_1}{n_1}$

$p=r_1+r_2$

$q=1-p$

We would like to express our thanks for the help given to us by several consultant histopathologists in Yorkshire in undertaking this work. In addition the Yorkshire Cancer Registry (Director, Professor C.A.F. Joslin) kindly provided their facilities and we would like to thank their staff and also the staff of the Yorkshire Regional Health Authority Regional Statistical Department for assistance. The funding for this study was provided by the Leeds Western District Special Trustees and the Yorkshire Cancer Research Campaign.

### References

BENNETT, M.H., FARRAR-BROWN, J., HENRY, K. & JELIFFE, A.M. (1974). Classification of non-Hodgkin's lymphoma. *Lancet*, **ii**, 405.

BERNARD, S.M., CARTWRIGHT, R.A., BIRD, C.C., RICHARDS, I.D.G., LAUDER, I. & ROBERTS, B.E. (1984). Aetiologic factors in lymphoid malignancies: a case-control epidemiology study. *Leukaemia Res.*, **8**, 681.

BIRD, C.C., LAUDER, I., KELLETT, H.S., CHORLTON, I., BARNES, N., DARWIN, C., CARTWRIGHT, R.A., BOYKO, R. (1984). Yorkshire Regional Lymphoma Histopathology Panel: Analysis of five years experience. *J. Path.*, **143**, 249.

LUKES, R.J. & BUTLER, J.L. (1966). The pathology and nomenclature of Hodgkin's disease. *Cancer Res.*, **26**, 1063.

NCI, Non-Hodgkin's Lymphoma classification project writing committee. (1985). Classification of Non-Hodgkin's Lymphomas. *Cancer*, **55**, 91.

OPCS. (1978–1985). Cancer statistics registrations: cases of diagnosed cancer registered in England and Wales 1968–80. Series MB1, Nos 1–13, HMSO.

WATERHOUSE, J., MUIR, C., SHANMUGARATRAM, K. & POWELL, J. (1982). Cancer incidence in five continents. IARC, WHO, Geneva, Switzerland.